# RE: The cost impact of unselective vs selective MammaPrint testing in early-stage breast cancer in Southern Africa

**DOI:** 10.1016/j.breast.2022.01.009

**Published:** 2022-01-22

**Authors:** Fabio Postiglione Mansani, Ruffo de Freitas-Junior

**Affiliations:** a Universidade Estadual de Ponta Grossa, Brazil; b Universidade Federal de Goias, Brazil

## Dear editor

Few studies have been conducted to assess the financial balance of the use of genetic signatures in breast cancer, as was published in this journal by Myburgh ([1]) with MammaPrint™ in Southern Africa. This analysis demonstrated a reduction in costs of 19.1% in patients with clinical high risk. Similar results were found in a pharmaco economic study carried out by us in Brazil ([2]), where the costs of chemotherapy drugs and MammaPrint™ were analyzed in the population at high clinical risk, where the reduction in the costs of adjuvant chemotherapy in this population exceeds USD $9600 per patient.

Both studies have limitations such as exchange rate variation, common in developing countries, which alters the costs of chemotherapy and genetic platform; also, because the indirect costs of the treatment were not considered, which would probably further increase the resource savings determined by the application of MammaPrint™. It is worth emphasizing the behavior of MammaPrint™ in different populations, which can determine different outcomes with the application of the test [[3], [4], [5]].

The analysis of the studies by Myburgh (1) and Mansani (2) show that the use of MammaPrint in the population at high clinical risk, in developing countries, results in significant savings in resources, which are often limited in these regions.

## Declaration of competing interest

I declare that receive funding from Agendia and GenCell Pharma for researching MammaPrint™ in the brazilian population.

## References

[1] Myburgh E.J., de Jager J.J., Murray E., Grant K.A., Kotze M.J., de Klerk H. (2021). The cost impact of unselective vs selective MammaPrint testing in early-stage breast cancer in Southern Africa. Breast Off J Eur Soc Mastology.

[2] Mansani F.P., Koppen M. (2020). Cancer research [internet].

[3] Piccart M., van’t Veer L., Poncet C., Cardozo J.M.N.L., Delaloge S., Pierga J. (2021). 70-Gene signature as an aid to treatment decisions in early breast cancer: updated results MINDACT. Lancet Oncol.

[4] Dubsky P., Van’t Veer L., Gnant M., Rudas M., Bago-Horvath Z., Greil R. (2021).

[5] Mansani F.P., Freitas-Junior R., SABCS (2021). https://cattendee.abstractsonline.com/meeting/10462/Presentation/939.

